# Signalling Pathways that Inhibit the Capacity of Precursor Cells for Myelin Repair

**DOI:** 10.3390/ijms14011031

**Published:** 2013-01-07

**Authors:** Jennifer K. Sabo, Holly S. Cate

**Affiliations:** Centre for Neuroscience Research, Department of Anatomy and Neuroscience, University of Melbourne, Melbourne Brain Centre, Kenneth Myer Building, 30 Royal Parade, Parkville, Vic 3010, Australia; E-Mail: jennifer.sabo@unimelb.edu.au

**Keywords:** oligodendrocyte progenitor cell (OPC), neural precursor cell (NPC), bone morphogenic protein (BMP), central nervous system (CNS)

## Abstract

In demyelinating disorders such as Multiple Sclerosis (MS), targets of injury are myelin and oligodendrocytes, leading to severe neurological dysfunction. Regenerative therapies aimed at promoting oligodendrocyte maturation and remyelination are promising strategies for treatment in demyelinating disorders. Endogenous precursor cells or exogenous transplanted cells are potential sources for remyelinating oligodendrocytes in the central nervous system (CNS). Several signalling pathways have been implicated in regulating the capacity of these cell populations for myelin repair. Here, we review neural precursor cells and oligodendrocyte progenitor cells as potential sources for remyelinating oligodendrocytes and evidence for the functional role of key signalling pathways in inhibiting regeneration from these precursor cell populations.

## 1. Introduction

In demyelinating diseases of the central nervous system (CNS), targets of injury are myelin and oligodendrocytes leading to a profound loss of myelin sheaths, axonal injury and degeneration. Demyelination occurs in a range of human neurological conditions, most notably in Multiple Sclerosis (MS) in adults and white matter injury in the newborn brain. In these demyelinating diseases, the body has a natural mechanism for repair by a process called remyelination, in which axons are provided with a new, thinner myelin sheath by the regeneration of mature oligodendrocytes [1]. There is evidence that remyelination can result in rapid functional recovery in animal models [2] and can be extensive in a proportion of MS patients in which the majority of lesions are remyelinated shadow plaques [3]. While remyelination occurs in MS, it is not complete and is characterized by shadow plaques, which have reduced myelin staining and are comprised of axons with thin myelin sheaths [4]. Remyelinated shadow plaques can be prone to subsequent bouts of inflammatory demyelination [5,6]. Remyelination failure has also been associated with axonal loss [7], which begins early in the disease [8] and is a major contributor to disability in patients [9]. In addition, Bramow *et al.* [6] observed incomplete remyelination in the spinal cord of progressive MS patients, which correlated with disease-related disability. Therefore, enhancement of endogenous remyelination is a highly promising approach for treatment of demyelinating diseases [1].

Signalling pathways have been identified that are inhibitory for oligodendrocyte differentiation and maturation and consequently for remyelination. Here, we review the evidence for these signaling pathways in regulating the response of oligodendrocyte progenitor cells (OPCs) and neural precursor cells (NPCs), which give rise to mature oligodendrocytes to promote remyelination. We summarize the roles of these signalling pathways in demyelination and remyelination and how they can be inhibited to promote myelin repair.

## 2. Endogenous Sources for Repopulation of Oligodendrocytes

### 2.1. Oligodendrocyte Progenitor Cells

Oligodendrocyte progenitor cells (OPCs) are an endogenous source for replacement of oligodendrocytes [10]. During development, OPCs are derived from ventricular zone precursor cells in the embryonic spinal cord and brain, with dorsal oligodendrocytes comprising only 15% of the total [11]. In the ventricular zone, there are ventral sources of OPCs, which are influenced by sonic hedgehog (Shh) signalling and express the transcription factors Nkx6.1 and Nkx6.2 [12]. Bone morphogenic protein (BMP) and fibroblast growth factor (FGF) signalling regulate the fate specification of dorsal OPCs [13,14]. Dorsal and ventral OPCs have similar electrophysiological properties, but differ in their migration patterns [15]. Ventral OPCs appear first in the spinal cord and spread throughout the white matter, whereas, dorsal OPCs arrived later and are restricted mainly to dorsal axon tracts [15]. The expression of the basic helix-loop-helix transcription factor, Olig2, is activated by the expression of Nkx6.1 and Nkx6.2 [16]. In turn, Olig2 induces the expression of the transcription factor Sox10 [17]. In response to Shh signalling, OPCs express Olig2 and platelet-derived growth factor receptor-α (PDGFRα) [18]. Olig2 plays a critical role in motor neuron and oligodendrocyte fate specification [19], and interacts with the transcription factor, Nkx2.2, to promote oligodendrocyte differentiation [20]. In the spinal cord of embryonic Olig2-null mice, there is an absence of oligodendrocytes, indicating that Olig2 is required for oligodendrocyte specification [19]. Similarly, there is an absence of mature oligodendrocytes in Nkx2.2-null mice and Sox10-null mice, suggesting the importance of these factors in oligodendrocyte maturation [21,22]. In contrast, oligodendrocyte maturation is delayed in Olig1-null mice; however, oligodendrocytes eventually develop [19].

Once the oligodendrocyte lineage is specified during development, the OPCs subsequently migrate and proliferate throughout the CNS [23–25]. Extracellular matrix molecules [26] and the chemokine, CXCL1 [27] regulate the migration of OPCs, while the presence of platelet-derived growth factor (PDGF) enhances their proliferative response [28]. In addition, insulin-like growth factor-1 (IGF-1) signalling plays a role in OPC proliferation during development [27]. At their final destination, OPCs stop and mature into myelinating oligodendrocytes dependent on the influence of axon-derived signals [29]. Mature, myelinating oligodendrocytes express MAG, myelin basic protein (MBP), proteolipid protein (PLP), myelin oligodendrocyte protein (MOG) and others [30].

The adult CNS also contains OPCs, indicating that not all OPCs differentiate during development [30,31]. In the developing CNS, OPCs express PDGFRα and Neuro/glial cell 2 chondroitin sulphate proteoglycan (NG2) [32], which continues into adulthood [31]. In the adult CNS, OPCs are located throughout the parenchyma [33] and genetic lineage tracing in transgenic mice has shown that OPCs are an endogenous source of mature, myelinating oligodendrocytes in the corpus callosum and cortical gray matter [31]. Patch clamping studies in the adult revealed there are two electrically distinct classes of OPCs, those that either express or lack voltage-gated sodium channels [34].

Oligodendrocytes regenerate naturally by differentiation of OPCs residing within the adult CNS in white and gray matter [35–39]. Experiments using *in vitro* techniques show that OPCs can be readily differentiated into mature oligodendrocytes [40], and in animal models of demyelination, retroviral labeling and genetic lineage tracing have shown that resident OPCs generate remyelinating oligodendrocytes within lesions [36,41]. The first step in the remyelination process depends on the OPCs responding to inflammatory stimuli arising from the secretion of factors by reactive astrocytes and microglia within demyelinated lesions [1,10]. Next, the OPCs must migrate to the lesion, proliferate and differentiate into remyelinating oligodendrocytes [1,10]. The transcription factors Nkx2.2 and Olig2 are expressed at high levels in OPCs within demyelinated lesions, suggesting an important role for these two genes in the process of OPC differentiation into remyelinating oligodendrocytes [42]. The expression of Nkx2.2 and Olig2, was also identified in OPCs in adult human CNS white matter brain tissue [43]. Kuhlmann *et al.* [43] reported that early human MS lesions expressed a higher number of OPCs and mature oligodendrocytes compared to chronic lesions, in which OPC numbers were lower and mature oligodendrocytes were rarely observed. These data suggest that some OPCs are present in chronic demyelinated lesions; however, there is an apparent failure of the OPCs to differentiate into mature oligodendrocytes and contribute to remyelination [43].

### 2.2. Neural Precursor Cells

Neural stem cells and multipotential neural precursor cells (NPCs) residing within the subventricular zone (SVZ), an active region of neurogenesis within the adult brain [44], could also provide a source to replace lost oligodendroglia [45,46]. The NPCs are thought to possess an unlimited proliferative capacity and produce neurons, oligodendrocytes, and astrocytes [47–49]. Under normal conditions, OPCs derived from SVZ cells in the adult mouse can migrate long distances into neighbouring white matter tracks and differentiate into mature oligodendrocytes [50].

In response to brain injury, cells produced in the SVZ have been shown to migrate to injured areas and differentiate into oligodendrocytes, astrocytes, and neurons [51]. There is evidence from two mouse models of demyelination (experimental autoimmune encephalomyelitis, which is a model of multifocal, inflammatory demyelination, and lysolecithin, which is a model of focal, toxic demyelination) that NPCs migrate from the SVZ into the demyelinated corpus callosum where they differentiate into oligodendrocytes [45,46,50,52,53]. Overexpression of Zfp488, the oligodendrocyte-specific zinc finger transcription repressor, increased the differentiation of SVZ NPCs into oligodendrocytes within the demyelinated corpus callosum and promoted functional recovery [54]. Moreover, in the corpus callosum, demyelinated axons form active glutamatergic synapses onto SVZ-born OPCs, which later give rise to mature oligodendrocytes [55]. In MS patients, Nait-Oumesmar *et al.* [56] reported an increase in PSA-NCAM+ progenitors in the SVZ, and in nearby lesions they detected PSA-NCAM+ cells that expressed the oligodendroglial markers, Olig2 and Sox10. The SVZ NPCs express the neurotrophin receptor, p75^NTR^, and Petratos *et al.* [57] showed an increase in p75^NTR^+ cells in the rodent and human MS SVZ. These data support the idea that endogenous adult SVZ precursors have the capacity to respond to demyelination and replace lost oligodendroglia [58].

## 3. Exogenous Sources for Repopulation of Oligodendrocytes

Cell transplantation represents a potential therapeutic strategy for the treatment of myelin disorders such as MS. Transplanted cells can include NPCs, which, following injection into the brain of hypomyelinated mice, can result in differentiation into oligodendrocytes and myelin production [59]. In animal models of demyelination, rodent NPC injections also contribute to remyelination of axons [60–62]. Pluchino *et al.* [60] injected NPCs intracerebroventricularly or intravenously into chronic EAE mice and showed that NPCs differentiated into OPCs which made close contact with thinly myelinated axons. Furthermore, using the chronic cuprizone-induced demyelination model (a model in which animals are fed the copper chelator cuprizone to induce a sustained toxic demyelination of the corpus callosum as well as other white matter tracts in the brain), NPCs were transplanted into the lateral ventricle of cuprizone-treated mice and induced the proliferation of endogenous NG2+BrdU+ cells, which the authors suggested contributed to enhanced remyelination in the caudal corpus callosum [61]. In a mouse viral infection model of inflammation and CNS demyelination (JHM strain of mouse hepatitis virus), NPCs transplanted into the spinal cord migrated to demyelinated areas, differentiated into oligodendrocytes and formed MBP+ myelin processes that made contact with axons [62]. Electron microscopy results showed there was an increase in the percentage of remyelinated axons in the spinal cord of mice with NPC transplants. Conversely, there was not enhanced remyelination in the spinal cord of mice transplanted with Olig1 knockout NPCs, in which the majority differentiated into GFAP+ cells, suggesting that Olig1 plays a role in the differentiation of NPCs to OPCs [62].

In recent studies, banked human CNS stem/precursor cells (HuCNS-SC) were successfully transplanted into the brains of hypomyelinated mice and patients with Pelizaeus-Merzbacher Disease (PMD), a rare congenital leukodystrophy in which developmental myelination is never established [63,64]. The HuCNS-SC transplanted into the hypomyelinated brains of neonatal and juvenile shiverer-immunodeficient mice migrated and preferentially differentiated into mature CC1+ oligodendrocytes in white matter, which generated mature, compact myelin with normal node formation and enhanced nerve conduction [63]. In a Phase 1 study, HuCNS-SC were transplanted into the brains of four male PMD patients with favourable safety results at 1 year after transplant [64]. Magnetic resonance imaging results suggested there was neural stem cell engraftment and transplant-derived myelin [64].

Human glial-restricted progenitors (GRPs) and OPCs have also been shown to generate donor-derived myelin in congenital hypomyelinated mice. Windrem *et al.* [65] transplanted fetal GRPs and adult OPCs into the forebrains of newborn shiverer mice with different levels of efficacy. Fetal GRPs transplanted at postnatal day 1 migrated into the forebrain white matter and differentiated into MBP+ oligodendrocytes and GFAP+ astrocytes after 12 weeks. At the ultrastructural level, graft recipients had dense, compact myelin sheaths compared to the loose myelin wrapping of typical shiverer axons [65]. On the other hand, adult OPCs differentiated into MBP+ oligodendrocytes 4 weeks after transplantation with ultrastructural evidence of compact myelin present in mice at 6 weeks [65]. In a subsequent study, immunocompromised neonatal shiverer mice were transplanted at multiple sites throughout the brain and spinal cord white matter tracts with human fetal GRPs [66]. After 1 year, the engrafted cells resulted in widespread, efficient myelination of a majority of axons throughout the CNS, with normal node formation and white matter comprised of greater than 30% human cells. Interestingly, some transplanted mice had an increased lifespan with improved neurological recovery [66].

While transplanted OPCs are effective in myelinating shiverer mice, treatment of MS patients with exogenous OPCs remains a challenge to the field to be viable as a potential therapy. In MS, lesions are multifocal and heterogeneous, in contrast to shiverer mice, which have global hypomyelination making evidence of donor-derived myelin very clear. In the MS brain, directly targeting lesions with exogenous OPCs would be difficult, given that lesions may need to be neuropathologically assessed to determine if they were predisposed to repair. For example, acute lesions are more prone to repair in the cuprizone model of demyelination than chronic lesions, where the infiltration of inflammatory cells is reduced compared to what is observed in acute lesions [67], suggesting an inflammatory environment may be critical for OPC differentiation and remyelination [68]. In addition, one would have to consider having adequate amounts of human OPCs for treatment and the migratory potential of these cells following transplantation. Hence, it would seem that treating MS patients with exogenous OPCs is premature, and that insights from neural stem transplantation in PMD patients could be a powerful basis for cell-based therapy in MS.

## 4. Signalling Pathways that Influence Myelin Repair

Here, we discuss signalling pathways that play central roles in the regulation of endogenous precursor cells and their capacity to inhibit myelin repair (Table 1). This review will mainly focus on the Wnt pathway, BMP pathway, Fibroblast growth factor 2 and Notch pathways.

### 4.1. Wnt Pathway

In the developing spinal cord, the Wnt proteins are dorsal factors that signal through the canonical β-catenin signalling pathway and inhibit the differentiation of oligodendrocytes [70,93]. In embryonic spinal cord explant cultures, Wnt-3a application decreased the number of O4+ cells, but did not affect the overall number of PDGFRα+ cells, whereas rmFz-8/Fc, a Wnt/β-catenin pathway inhibitor, increased the number of O4+ cells, indicating an role for endogenous Wnt proteins in oligodendrocyte differentiation [93]. In rat OPC cultures, exogenous Wnt3a decreased the number of GalC+ oligodendrocytes, while there was no change in the number of A2B5+ OPCs [70]. In addition, transgenic mice where β-catenin is constitutively active in oligodendrocyte lineage cells showed decreased numbers of PLP+ oligodendrocytes and myelinated axons in the postnatal spinal cord, however, normal levels were reached by adulthood [70]. Taken together, these results suggest that Wnt signalling is inhibitory for OPC differentiation.

The Wnt pathway has been shown to be active during developmental myelination and in remyelination in the adult. The transcription factor Tcf4, a mediator of Wnt signalling, forms a complex with β-catenin in the nucleus and regulates the expression of target genes [94]. Fancy *et al.* [69] showed that Tcf4 was expressed in OPCs during developmental myelination at postnatal day 1 and postnatal day 15 in the healthy rodent spinal cord and within remyelinating lesions of the lysolecithin-demyelinated spinal cord. In human developing white matter and active MS lesions, there was a similar expression pattern of Tcf4 in oligodendrocyte lineage cells [69]. *In vivo* overexpression of β-catenin in Olig2+ cells in mice resulted in developmental hypomyelination due to an OPC differentiation delay, which was resolved by adulthood. When these mice were exposed to focal demyelination of the spinal cord, there was no change in OPC numbers, however, there was a decrease in the number of PLP+ oligodendrocytes and impaired remyelination, indicating that a delay in OPC differentiation contributed to a lack of repair [69]. Furthermore, Ye *et al.* [95] provided evidence that two histone modifying enzymes, HDAC1 and HDAC2 antagonize the inhibition of Wnt signaling on oligodendrocyte differentiation and that Tcf4 (TCF7L2) mediates this crosstalk. More recently, Fancy *et al.* [71] have shown that delivery of XAV939, a Wnt antagonist, into spinal cord demyelinated lesions in mice, accelerated the differentiation of Nkx2.2+ OPCs into mature PLP+ oligodendrocytes and promoted remyelination *in vivo*. These studies provide evidence that the Wnt pathway is an antagonist of myelination and remyelination *in vivo*.

### 4.2. BMP Pathway

The roles of Bone Morphogenic Proteins (BMPs) during development have been very well characterised, however, their role within the adult brain is less well described, particularly in the context of a demyelinating insult. There is recent evidence that BMP signalling influences the response of progenitor cells *in vivo* within the adult SVZ and in the demyelinated lesion following cuprizone-induced demyelination in mice [72,73]. It has been shown that BMP signalling influences the commitment of SVZ cells to the oligodendrocyte lineage [53,72,75] and is tightly regulated by the inhibitor, Noggin, which is expressed in the ependymal cells of the SVZ [96]. Previous work in our laboratory has shown that inhibiting BMP signalling by Noggin infusion into the lateral ventricles in mice increases numbers of Olig2+ oligodendroglia and decreases numbers of GFAP+ astrocytes in the SVZ during demyelination [72]. In addition, Jablonska *et al.* [53] found that Chordin, a BMP antagonist with slightly lower affinity for BMP4 than Noggin [97], is upregulated in the SVZ during demyelination. In this study, infusion of Chordin induced the migration of DCX-expressing cells from the SVZ into the demyelinated corpus callosum where they differentiated into oligodendrocytes [53]. It is currently unclear whether Noggin also alters the migration of SVZ cells into the demyelinated lesion and their subsequent differentiation into mature oligodendrocytes.

There is an important role for BMP signalling in modulating differentiation and remyelination from endogenous OPCs within cuprizone-induced demyelinated lesions of the mouse brain. Intraventricular infusion of BMP4 into the brains of mice during cuprizone-induced demyelination revealed that exogenous BMP signalling transiently increases the proliferation of endogenous OPCs that are rapidly cleared following recovery from a demyelinating insult [73]. In contrast, infusion of Noggin, which inhibits endogenous BMP signalling during demyelination, promotes differentiation of OPCs into mature oligodendrocytes and myelin repair [73]. The relative contribution from resident OPCs and SVZ NPCs to myelin repair following Noggin infusion has yet to be determined.

In other animal models of white matter injury, there is evidence to suggest that downregulation of BMP signalling increases oligodendrogliogenesis and repair. Dizon *et al.* [98] reported that transgenic overexpression of Noggin increases Olig2+ cells in the corpus callosum following perinatal hypoxic-ischemic brain injury. Wang *et al.* [99] showed that reactive astrocytes in injured spinal cord express high levels of BMPs and that exogenous Noggin blocks the ability of reactive astrocytes and their conditioned media to inhibit differentiation of spinal cord OPCs into oligodendrocytes. Similarly, Noggin infusion following intraventricular hemorrhage enhances oligodendroglial maturation and myelination [100]. Additionally, Noggin treatment alleviates the neurological deficit associated with intraventricular hemorrhage [100]. These studies identify a function for BMP inhibition in enhancing repair following white matter injury.

Recent data from Weng *et al.* [76] support a role for BMP signalling in the inhibition of oligodendrocyte differentiation *in vivo* and provide insight into possible mechanisms for this action. Their data suggest that the transcription factor Smad-interacting protein-1 (Sip1) promotes oligodendrocyte maturation by inhibiting the BMP-SMAD pathway, by activating the expression of an inhibitory SMAD, SMAD7 [76]. Weng *et al.* [76] identified Sip1 as a common target gene of Olig1 and Olig2 and showed that conditional deletion of Sip1 in oligodendrocyte lineage cells *in vivo* results in an absence of myelin gene expression and myelination. Using coimmunoprecipitation and chromatin immunoprecipitation assays, Weng *et al.* [76] showed that Sip1 interacts with phosphorylated SMAD1 in a complex with SMAD4 and p300 and regulates the expression of oligodendrocyte differentiation inhibitors Id2 and Id4.

### 4.3. Fibroblast Growth Factor 2

Fibroblast growth factor 2 (FGF2) is a factor that plays an important role in the production of oligodendrocytes originating from NPCs in the healthy adult SVZ. NPCs in the adult mouse SVZ express FGF2, and FGF2 knockout mice have reduced numbers of slow-dividing NPCs, implicating a role for FGF2 in maintenance of NPCs [77]. Azim *et al.* [83] recently showed that intraventricular delivery of FGF2 into the brain of postnatal mice expanded the population of Nestin+ and PDGFRα+ cells in the SVZ and proliferating OPCs in the corpus callosum. Thus, FGF2 appears to be important for the maintenance and fate of SVZ cells.

In the mouse spinal cord, FGF2 mRNA and protein expression is increased from postnatal day 7 to postnatal day 15, spanning the postnatal period of mature oligodendrocyte generation and myelination [78]. Knockdown of fibroblast growth factor receptor 1 (FGFR1) by RNA interference in OPC cultures promoted the differentiation of OPCs into O1+ oligodendrocytes, and *in vivo* retroviral infection of mouse postnatal day 7 spinal cord with a dominant negative form of FGF receptor increased OPC differentiation into mature CC1+ oligodendrocytes at postnatal day 28 [79]. Likewise, in the spinal cord of FGF2 knockout mice, retroviral labelling at postnatal day 7 revealed that a greater proportion of GFP+ cells were identified as mature oligodendrocytes expressing CC1 at postnatal day 28, indicating a role for endogenous FGF2 in the inhibition of OPC differentiation [78]. In adult rats, FGF2 intraventricular infusion resulted in the appearance of abnormal oligodendrocytes and myelin loss in the anterior medullary velum, which coincided with the appearance of NG2+ OPCs [101]. Likewise, examination of the corpus callosum after intraventricular injection of FGF2 *in vivo* increased the number of mature PLP+ and CC1+ oligodendrocytes and decreased myelination during development and in the adult [83]. The results from these studies highlight an inhibitory role for FGF2 in the differentiation of oligodendrocyte lineage cells.

FGF2 plays a key role within the corpus callosum during cuprizone-induced demyelination and remyelination. In acute demyelinated lesions induced by cuprizone challenge, FGF2 mRNA expression is increased in the corpus callosum [80]. Furthermore, prolonged oral administration of cuprizone for up to 12-weeks can result in chronic demyelinated lesions in the corpus callosum where FGF2 mRNA expression is also increased [67,81]. In FGF2 knockout mice, there was an increase in the density of PLP+ oligodendrocytes, and evidence from myelin staining indicated enhanced remyelination following recovery from acute and chronic cuprizone challenge [80,81]. In chronic demyelinated lesions, electron microscopy analysis of the caudal corpus callosum revealed spontaneous remyelination, and immunohistochemical analysis of neurofilament showed there was reduced axonal damage in FGF2 knockout mice compared to wildtype mice [82]. Taken together, these studies suggest that FGF2 is a negative regulator of remyelination in the context of acute and chronic cuprizone-induced demyelination.

While several studies have reported that genetic deletion of FGF2 is beneficial for remyelination *in vivo* where the blood-brain barrier is intact during demyelination [80–82], there are studies to suggest a neuroprotective role for FGF2 in inflammatory-mediated demyelination. In chronic EAE mice, intracisternal injection of the FGF2 gene after disease induction ameliorated clinical symptoms [84]. In addition, Ruffini *et al.* [84] reported decreased demyelination and axonal loss and increased numbers of PDGFRα+ OPCs and PLP+ oligodendrocytes in the spinal cord of FGF2-treated EAE mice after 60 days. Furthermore, the clinical symptoms of chronic EAE were worsened in FGF2 knockout mice, which had increased numbers of CD8+ T cells and macrophages/microglia in spinal cord lesions, which electron microscopy revealed to have diminished numbers of remyelinated axons [102]. Thus, the role of FGF2 in remyelination appears to be dependent on the animal model of demyelination and the immune response.

Levels of FGF2 have also been evaluated in human MS patients. In active demyelinating plaques and chronic active and inactive plaques from the brains of MS patients, Clemente *et al.* [85] described an upregulation of FGF2 in macrophages/microglia, in lesion areas predisposed to remyelination, whereas FGFR1, the receptor of FGF2, was expressed in PDGFRα+ OPCs in chronic active lesions, which the authors suggest could be recruited in response to FGF2. Moreover, FGF2 levels were evaluated in the cerebrospinal fluid of MS patients and were found to be significantly higher than in control patients [86]. Both studies suggest that FGF2 could be implicated in the pathogenesis of MS.

### 4.4. Notch Pathway

During development, the Notch pathway, which is mediated by the Jagged and Delta ligands, regulates OPC differentiation and myelination. In cultures from postnatal rat optic nerve, OPCs, oligodendrocytes and astrocytes express the Notch1 receptor while its ligand, Jagged1, is expressed by oligodendrocytes and retinal ganglion cells [103]. When OPCs are co-cultured with Jagged1 expressing cells or in conditioned medium containing soluble Delta1, OPCs fail to differentiate and remain A2B5+ [103]. In another study, primary human oligodendrocytes expressing O4 and CNPase (2′,3′-Cyclic-nucleotide 3′-phosphodiesterase) and not expressing MBP were transfected with Jagged1 and differentiated into MBP+ cells with very few processes, indicating an inhibitory effect of Jagged1 on oligodendrocyte maturation [92]. In rat OPC cultures, knockdown of Notch1 using siRNA inhibited OPC proliferation and enhanced OPC differentiation into O4+, CNPase+ and MBP+ oligodendrocytes and myelin segment formation [89]. Furthermore, conditional deletion of Notch1 in oligodendrocytes *in vivo* resulted in precocious differentiation of premyelinating oligodendrocytes in the spinal cord [87,89]. Notch1 heterozygous knockout mice showed increased myelination in the brain at postnatal day 15 and numbers of myelinated axons in the optic nerve at postnatal day 35 [88]. These findings indicate that activation of the Notch pathway is inhibitory for OPC differentiation and myelination.

Demyelinating and remyelinating lesions have been shown to express members of the Notch signalling pathway. In the demyelinated spinal cord of EAE animals, Notch1 was expressed in macrophages and astrocytes, while Jagged1 expression was restricted to astrocytes [90]. However, in fully remyelinated lesions from EAE animals, oligodendrocytes were the most abundant cellular source of Notch1 [90]. Furthermore, Stidworthy *et al.* [91] examined the expression profile of Notch1 and Jagged1 during the remyelination following focal demyelination induced by ethidium bromide in the brain. In this study, Notch1 was predominantly expressed by NG2+ OPCs during remyelination, while there were multiple cellular Jagged 1 sources, which did not include demyelinated axons [91]. These studies suggest that Notch signalling could play a role during demyelination and remyelination in animal models.

Studies using genetic deletion strategies have also been used to describe the effect of Notch signalling on remyelination. Zhang *et al.* [89] examined remyelination of the lysolecithin-demyelinated corpus callosum in mice where Notch1 was inactivated in oligodendrocyte lineage cells using Olig1Cre:Notch1^2f/12f^ mice. In these mice, there was a reduction in the area of the demyelinated lesion and in OPC proliferation compared to controls, and electron microscopy analysis revealed an increase in the percentage of axons with high g ratios (*i.e.*, the ratio between the axon diameter and fiber diameter as measure of myelin thickness), suggesting that remyelination was enhanced. This evidence suggests Notch1 signalling regulates OPC differentiation in the context of myelin repair. However, in another study, Stidworthy *et al.* [91] showed that conditional deletion of Notch1 in PLP+ oligodendrocytes *in vivo* did not alter remyelination in the corpus callosum after cuprizone-induced demyelination. The authors interpret this as an indication that Notch signalling is not a rate-determining factor during remyelination.

The Notch pathway has also been investigated in human MS lesions. In adjacent sections from an acute lesion, John *et al.* [92] showed that Jagged1 was expressed in hypertrophic GFAP+ astrocytes, whereas Notch1 and Hes5 (a downstream Notch target gene which inhibits oligodendrocyte differentiation [104]) were expressed in immature oligodendrocytes. In addition, Jagged1 was found to be differentially expressed in actively demyelinated and remyelinated lesions in the same brain tissue. Jagged1 was strongly expressed in hypertrophic astrocytes within the center and rim of the demyelinated lesion, while there were few Jagged1+ cells in the adjacent normal appearing white matter and no expression in remyelinated lesions [92]. The results suggest that Notch signalling is active in within human MS lesions and inhibitory for myelin repair.

## 5. Conclusions

Regenerative therapies aimed at promoting oligodendrocyte maturation and remyelination are promising strategies for treatment in CNS demyelinating diseases. Here we have reviewed some of the potential sources for remyelinating oligodendrocytes and evidence for the functional role of four signalling pathways (Wnt pathway, BMP pathway, Fibroblast growth factor 2 and Notch pathways) in inhibiting regeneration from these precursor cell populations. We have provided evidence that these pathways are inhibitory for OPC differentiation and maturation. Furthermore, we have reviewed published work that suggests inhibition of these pathways promotes mature oligodendrocytes and/or remyelination. Manipulation of some or all of these pathways *in vivo* is likely to be important in providing a lesion environment conducive to repair and should be considered in strategies to promote recovery from endogenous precursor cells or through the use of cell-based therapies with the ultimate aim to provide therapeutic benefit in the treatment of demyelinating disorders.

## Figures and Tables

**Table 1:** Signalling pathways that regulate the response of endogenous precursor cells and the implications for myelin repair.

Signalling Pathway	Regulation of Endogenous NPC	Regulation of Endogenous OPC	Capacity for Myelin Repair
Wnt pathway	-	Inhibitory for OPC differentiation during developmental myelination *in vivo* (↓)[69,70]	Tcf4 and Axin2 are expressed in oligodendroglia in remyelinating lesions in rodents and in active MS lesions and neonatal white matter lesions in humans [69,71]
			*In vivo* delivery of a Wnt pathway antagonist promoted OPC differentiation and remyelination in the spinal cord of lysolecithin-demyelinated mice (↑) [71]

BMP pathway	Inhibiting BMP signalling with Noggin infusion during cuprizone-induced demyelination increased oligodendroglial cell numbers in the SVZ (↑) [72]	Increasing BMP signalling during cuprizone-induced demyelination increased OPC proliferation within lesions (↑) [73]	Downregulation of endogenous BMP signalling during demyelination promoted mature oligodendrocyte regeneration and myelin repair (↑) [73]
	Inhibiting BMP signalling with Chordin infusion induced the differentiation of SVZ neuroblasts into oligodendrocytes in lysolecithin-demyelinated corpus callosum (↑) [53]	Inhibiting BMP signalling during cuprizone-induced demyelination increased OPC differentiation (↑) [73]	BMPs have been detected in human brain chronic MS lesions [74]
	Inhibiting BMP signalling with Noggin infusion increased numbers of Olig2+ cells in the adult healthy SVZ (↑) [75]	Transcription factor, Sip1, inhibits BMP signalling and promotes oligodendrocyte maturation *in vivo* (↑) [76]	

FGF2	FGF2 regulates SVZ NSC maintenance (↑) [77]	Inhibitory for OPC differentiation during developmental myelination (↓) [78,79]	Knockout mice show enhanced remyelination following recovery from acute and chronic cuprizone-induced demyelination (↑) [80–82]
	*In vivo* delivery increased numbers of SVZ OPCs (↑) [83]		Treatment of EAE mice increased OPC and mature oligodendrocyte numbers in the spinal cord (↑) [84]
			Upregulated in macrophages/microglia in human MS plaques [85] and cerebrospinal fluid of MS patients [86]

Notch pathway	-	Inhibitory for OPC differentiation *in vivo* (↓) [87–89]	Expressed in demyelinated and remyelinated lesions in animal models [90,91]
			Genetic deletion of Notch1 in oligodendrocytes enhanced remyelination of the lysolecithin-demyelinated spinal cord (↑) [89]
			Expressed in human demyelinated lesions, but absent in remyelinated lesions [92]
